# Mapping Heterogeneity in Psychological Risk Among University Students Using Explainable Machine Learning

**DOI:** 10.3390/e28020224

**Published:** 2026-02-14

**Authors:** Penglin Liu, Ji Tang, Hongxiao Wang, Dingsen Zhang

**Affiliations:** College of Information Science and Engineering, Northeastern University, Shenyang 110819, China

**Keywords:** TreeSHAP, psychological risk heterogeneity, Gaussian Mixture Models, student mental health, mechanism-based subtyping

## Abstract

In the post-pandemic era, student mental health challenges have emerged as a critical issue in higher education. However, conventional assessment approaches often treat at-risk populations as a monolithic entity, thereby limiting intervention effectiveness. This study proposes a novel computational framework that integrates explainable artificial intelligence (XAI) with unsupervised learning to decode the latent heterogeneity of psychological risk mechanisms. We developed a “predict-explain-discover” pipeline leveraging TreeSHAP and Gaussian Mixture Models to identify distinct risk subtypes based on a 2556-dimensional feature space encompassing lexical, linguistic, and affective indicators. Our approach identified three theoretically-grounded subtypes: academically-driven (28.46%), socio-emotional (43.85%), and internal regulatory (27.69%) risks. Sensitivity analysis using top-20 core features further validated the structural stability of these mechanisms, proving that the subtypes are anchored in the model’s primary decision drivers rather than high-dimensional noise. The framework demonstrates how black-box classifiers can be transformed into diagnostic tools, bridging the gap between predictive accuracy and mechanistic understanding. Our findings align with the Research Domain Criteria (RDoC) and establish a foundation for precision interventions targeting specific risk drivers. This work advances computational mental health research through methodological innovations in mechanism-based subtyping and practical strategies for personalized student support.

## 1. Introduction

Since the formal conclusion of the COVID-19 pandemic [1,2], the mental health landscape in higher education has undergone a tectonic shift, with student distress escalating in both prevalence and complexity [3,4]. This crisis has transformed student psychological well-being into a paramount global public health priority [5,6], placing unprecedented strain on university counseling infrastructures. While the rapid advancement of machine learning has yielded powerful tools for early risk screening [7,8], the prevailing research trajectory remains heavily skewed toward the optimization of predictive benchmarks [9,10,11]. Existing models excel at binary classification—effectively “flagging” students based on textual indicators—yet they often stop at the threshold of actionable insight.

The fundamental limitation of this “black-box” predictive paradigm lies in its treatment of the “high-risk” population as a monolithic entity. This approach inadvertently overlooks the profound phenotypic and etiological heterogeneity inherent in psychological distress [12,13]. In clinical reality, two students categorized as “high-risk” may exhibit identical risk scores but possess entirely divergent underlying drivers—one rooted in academic burnout and the other in chronic social isolation [14]. Treating these distinct profiles as a single group not only obscures the nuanced reality of student distress but also fundamentally [15,16,17] constrains the development of targeted, precision interventions. This challenge aligns with the Research Domain Criteria (RDoC) initiative, which advocates for a multidimensional understanding of psychological dysfunction rather than broad symptom-based categories [18].

Bridging this gap requires addressing the notorious trade-off between model performance and interpretability. Sophisticated ensemble learners, such as XGBoost, achieve superior accuracy by capturing high-order feature interactions, yet their decision-making logic remains opaque [14]. While game-theoretic frameworks like SHAP [16] provide a mathematical basis for quantifying feature importance, they are predominantly utilized for post-hoc validation. However, a significant opportunity exists to repurpose SHAP as a heuristic engine for scientific discovery. By analyzing the “decision geometry” within a 2556-dimensional attribution space, we can identify clusters of individuals who share similar causal-logic patterns as perceived by a high-performing model.

**Figure 1:**
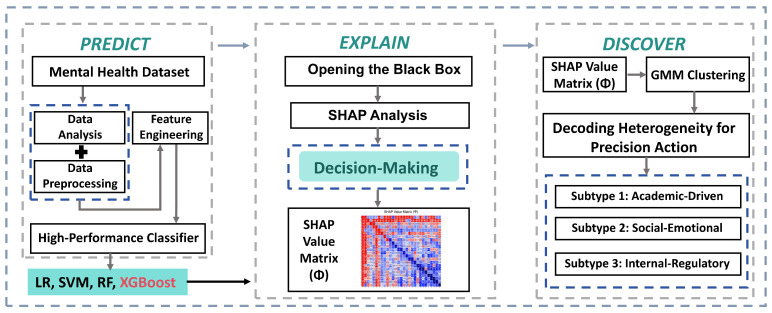
Predict-Explain-Discover Framework. This schematic illustrates our three-stage analytical pipeline. Stage 1 (Predict): Raw student text data undergoes comprehensive feature engineering and is used to train and evaluate a suite of machine learning models. The best-performing model (XGBoost) is selected based on its predictive accuracy. Stage 2 (Explain): The high-performing, yet opaque, “black-box” model is interpreted using SHapley Additive exPlanations (SHAP), which deconstructs its predictions into a matrix of feature contribution values (the SHAP value matrix, Φ). Stage 3 (Discover): This SHAP matrix, representing the model’s decision logic, is clustered using a Gaussian Mixture Model (GMM), revealing three distinct student subtypes. Each subtype is linked to a targeted intervention strategy, enabling a shift from one-size-fits-all to precision support.

In response, this study proposes a novel “Predict-Explain-Discover” framework (Figure 1) designed to decode the latent heterogeneity within high-risk populations. Grounded in the tripartite model of student distress—which categorizes risks into academic, socio-emotional, and internal regulatory domains [19]—our framework shifts the focus from raw data clustering to attribution-based phenotyping. We pose a central research question: Can we leverage the explanatory power of TreeSHAP to “reverse-engineer” a classifier’s logic, thereby identifying distinct, theoretically-grounded risk profiles? By doing so, we aim to transform the classifier from a simple gatekeeper into a diagnostic lens, facilitating a transition to personalized, mechanism-aware support strategies [20].

The principal contributions of this study are threefold:

1. Methodologically, we constructed an analytical pipeline that integrates TreeSHAP explanations with Gaussian Mixture Model (GMM) clustering, enabling the deconstruction of model decision-making across lexical, linguistic, and affective feature dimensions.

2. Theoretically, we identified three distinct mental risk subtypes—Academic-Driven, Socio-Emotional, and Internal Regulatory—demonstrating that these profiles remain structurally robust even under core-feature sensitivity analysis (Top-20 SHAP features).

3. Practically, we derived tailored intervention strategies for each subtype, facilitating a critical shift in student mental health management from merely predicting risk to precisely addressing its specific predictive drivers.

## 2. Theoretical Analysis

### 2.1. Supervised Learning: Robustness and Classification Logic

The foundational layer of our framework identifies at-risk individuals by transforming unstructured student texts into a 2556-dimensional feature space, comprising lexical (TF-IDF), linguistic, and affective indicators. We formulate this as a supervised learning task [2,21], where the objective is to learn a mapping function that distinguishes high-risk profiles from the baseline population. To ensure the reliability of our risk identification, we implemented a competitive evaluation across three distinct architectures: Logistic Regression (LR), Support Vector Machines (SVM), and XGBoost. While LR and SVM provide essential benchmarks for linear and margin-based separation, XGBoost was selected as our primary backbone due to its superior capacity to handle the sparse and imbalanced nature of psychological text data. Its objective function integrates a second-order Taylor expansion with a regularization term $\Omega (f_{t})$ to penalize model complexity:

$$L^{\left(t\right)}≃\sum_{i=1}^{n}\left[g_{i}f_{t}\left(x_{i}\right)+\frac{1}{2}h_{i}f_{t}^{2}\left(x_{i}\right)\right]+\gamma T+\frac{1}{2}{\lambda ||w||}^{2}$$

This regularization is critical for ensuring that the subsequent “discovery” phase is based on generalized psychological patterns rather than noise-specific over-fitting. The model was trained on a 650-sample augmented dataset with parameters finalized via 5-fold cross-validation.

### 2.2. Subtype Discovery: Transitioning to the Attribution Space

A major methodological shift in this study is the move from raw data clustering to attribution-based phenotyping [22]. We argue that clustering raw symptoms often yields fragmented results because the same behavior can be driven by divergent psychological stressors. By clustering in the SHAP attribution space, we group students based on the predictive weight the model assigns to their unique profiles.

Specifically, we employed TreeSHAP [16], an additive feature attribution method optimized for tree-based models, to decompose the classifier’s output. For each student in the test set (n=130), this yields a SHAP vector Φ, representing how features pull the risk probability away from the base rate. To identify latent subtypes, we utilized Gaussian Mixture Models (GMM). Unlike “hard” clustering methods like K-means, GMM is a probabilistic approach that models the population as a mixture of *K* multivariate Gaussian distributions:

$$p\left(\Phi \right)=\sum_{k=1}^{K}\pi_{k}N\left(\Phi |\mu_{k},\Sigma_{k}\right)$$

This approach is theoretically superior for psychological data, as it accounts for the “fuzzy” and overlapping nature of mental health states. To ensure a parsimonious selection, we utilized the Bayesian Information Criterion (BIC) to determine the optimal K=3. Furthermore, we conducted a sensitivity analysis using the Top-20 SHAP features to verify that the identified subtype structure remains robust when the input is restricted to the most influential predictive drivers.

### 2.3. Methodological Validation via Mutual Information

To bridge the gap between machine learning outputs and psychological theory, we utilized Mutual Information (MI) as a validation metric [23,24]. The goal is to quantify the “informational dependency” between our discovered clusters and the original feature set. Rather than relying on simple linear correlations, we employed the KSG estimator to capture non-linear relationships:

$$I\left(X;Y\right)=\psi \left(k\right)-\langle \psi \left(n_{x}+1\right)+\psi \left(n_{y}+1\right)\rangle +\psi \left(N\right)$$

High MI values indicate that a specific cluster (e.g., the “Academic-driven” subtype) is robustly characterized by a distinct set of features. This step serves as a mathematical filter, ensuring that the labels we assign to the subtypes—Academic, Socio-emotional, and Internal Regulation—are deeply rooted in statistical evidence. This provides a theoretically sound bridge to the tripartite model of student distress [19], aligning our data-driven findings with the RDoC initiative [18] and transforming the classifier into an interpretable diagnostic lens.

## 3. Methodology

### 3.1. Experimental Preparation

#### 3.1.1. Dataset Description

We used a public dataset from Kaggle consisting of 500 vocational education students. The dataset was generated/simulated to reflect realistic conditions for developing and testing AI-driven mental health support systems. Each record corresponds to one student and includes demographic attributes (age, gender), academic performance (GPA), mental health scores (stress, anxiety, depression), behavioral indicators (sleep hours, daily steps), and textual inputs (daily reflections and mood descriptions). A sentiment score was provided and derived using VADER analysis.

To improve model stability, we augmented the original cohort (N = 500) to N = 650 samples using text-based augmentation. Our preprocessing pipeline incorporated text cleaning and normalization, tokenization, and a multi-layered feature engineering approach. The final feature space consists of 2556 dimensions, comprising 2540 TF-IDF lexical features (min_df = 2, max_df = 0.95), 11 structural linguistic features, and 5 lexicon-based sentiment indicators.

#### 3.1.2. Benchmark Model Establishment

We implemented six representative classifiers to establish performance baselines: Logistic Regression, SVM, Random Forest, XGBoost, LightGBM, and Naïve Bayes. Each was evaluated using 5-fold cross-validation on the training set (n=520). Performance was assessed via F1-score and AUC-ROC to ensure a balance between sensitivity and precision. Comparative analysis identified XGBoost as the primary backbone for subsequent interpretability analysis due to its superior handling of the sparse, high-dimensional textual feature space.

### 3.2. Constructing a Heterogeneity Interpretation Framework

#### 3.2.1. Theoretical Foundation: TreeSHAP Attribution

The core innovation lies in transforming classifiers into discovery tools via explainable AI. We employ TreeSHAP [25], a variant of SHapley Additive exPlanations [26] optimized for tree-based ensembles. For a model *f* and input *x*, the SHAP model *g* satisfies:

(1) $$g\left(z^{\prime }\right)=\varphi_{0}+\sum_{i=1}^{M}\varphi_{i}z^{\prime }i$$

where $\varphi_{i}$ represents the Shapley value for feature *i*, calculated based on the conditional expectation of the model output. The resulting SHAP vector $\Phi \left(x\right)=\left[\varphi_{1},\;\varphi_{2},\ldots ,\;\varphi_{2556}\right]$ characterizes the specific decision logic assigned by the model to each student’s profile.

#### 3.2.2. Key Insight: Attribution-Based Phenotyping

Our fundamental premise is that students sharing similar patterns of decision logic—as encoded in their SHAP vectors—share similar underlying risk mechanisms. This represents a paradigm shift from traditional symptom-based clustering to attribution-based phenotyping [27]. Unlike raw features which capture superficial behaviors, SHAP values quantify the “predictive weight” of those behaviors, allowing our framework to group students by the internal logic that leads to their risk classification.

#### 3.2.3. Implementation Framework

The heterogeneity decoding framework comprises three systematic phases:

Phase 1: SHAP Matrix Generation.

We computed TreeSHAP values for all instances in the test set (n=130), generating a SHAP matrix $\Phi_{\mathrm{test}}\in R^{130\times 2556}$.

Phase 2: Subtype Discovery via Probabilistic Clustering.

We applied Gaussian Mixture Models (GMM) to the SHAP matrix, modeling the population as a mixture of *K* multivariate Gaussian distributions. This soft-clustering approach is theoretically superior for psychological data as it accounts for overlapping mental states. The optimal number of clusters, K=3, was determined through the Bayesian Information Criterion (BIC), ensuring an optimal trade-off between model complexity and the capture of psychological heterogeneity.

Phase 3: Robustness and Validation.

We validated the subtypes by:

(1) performing a sensitivity analysis using the Top-20 SHAP features to confirm that the cluster structure remains stable when restricted to core predictive drivers;

(2) utilizing t-SNE for visualization to inspect the decision manifold (noting that t-SNE serves as an exploratory tool rather than structural evidence);

(3) employing Mutual Information (MI) analysis to quantify the informational dependency between original features and cluster assignments.

#### 3.2.4. Methodological Advantages

**Figure 2:**
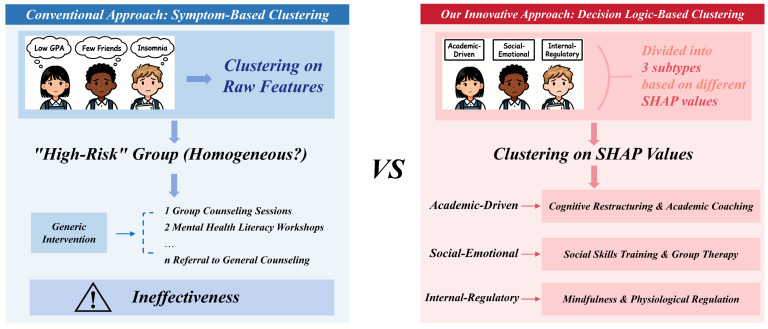
**Left** (Conventional Approach): Students are grouped based on co-occurring surface-level symptoms in their raw data (e.g., low GPA, few friends, insomnia). This symptom-based clustering results in a single, heterogeneous “high-risk” group, leading to generic, one-size-fits-all interventions that fail to address the root causes. **Right** (Our Approach): Students are grouped based on the underlying decision logic of the predictive model (i.e., their SHAP value profiles). This mechanism-based clustering clearly differentiates students into three distinct subtypes, each defined by a unique combination of driving factors (e.g., high academic pressure vs. high loneliness vs. poor sleep quality). This precise subtyping directly informs the development of tailored, precision interventions.

As illustrated in Figure 2, our framework transcends superficial symptom clustering by focusing on the actual decision mechanisms driving risk classification. By integrating TreeSHAP with GMM, we enable the discovery of theoretically-grounded risk profiles—Academic, Socio-emotional, and Internal Regulatory—that remain obscured in traditional “black-box” methodologies.

This approach represents a novel integration of explainable AI and unsupervised learning, transforming predictive models into discovery tools that effectively address the critical gap between predicting risk and understanding the underlying heterogeneous mechanisms [28].

## 4. Experiments

### 4.1. Experimental Design and Setup

#### 4.1.1. Data Preprocessing

**Figure 3:**
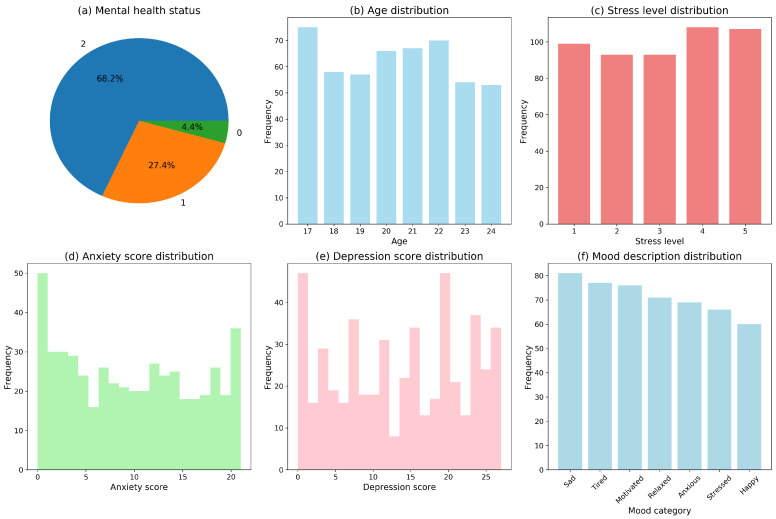
Mental-health Dataset Visualization Summary. (**a**) Mental health status: the pie chart depicts that a majority of students experience mild-to-moderate psychological distress, with only a minority being completely asymptomatic. (**b**) Age distribution: the histogram shows a concentration of participants aged 17–23 years, indicating a sample composition predominantly comprising first- to third-year undergraduates. (**c**) Stress level distribution: the histogram exhibits a multimodal or platykurtic pattern, suggesting considerable variability in stress manifestations, with a subgroup clustering in the high-score range. (**d**) Anxiety score distribution: a similarly heterogeneous profile is observed, reinforcing the diversity in anxiety-related symptoms across the population. (**e**) Depression score distribution: the broad and flattened distribution implies varying degrees of depressive symptoms, with noticeable aggregation at elevated severity levels. (**f**) Mood description distribution: the bar chart identifies “sadness, fatigue, and lack of motivation” as the most frequently endorsed emotional states, reflecting a generally negative affective tone in daily experiences.

As illustrated in Figure 3, the raw dataset exhibits pronounced distributional heterogeneity across age, stress levels, anxiety/depression scores, and emotional categories, alongside a moderate degree of imbalance in the mental health labels. In response to these characteristics, the data preprocessing pipeline systematically addressed missing value imputation, outlier detection and handling, categorical variable encoding, and numerical feature standardization. These procedures were implemented to ensure statistical robustness and to provide stable, well-conditioned inputs for subsequent classification modeling and interpretability analyses.

#### 4.1.2. Experimental Configuration

Experiments were conducted using a 5-fold cross-validation scheme. The 650 samples were partitioned into a training set of 520 instances and a test set of 130 instances (Table 1), with the random seed fixed at 42 to ensure reproducibility. Hyperparameter optimization was conducted via grid search for all models. Evaluation metrics included accuracy, precision, recall, F1-score, and AUC-ROC, providing a multi-faceted assessment of classification performance.

### 4.2. Benchmark Model Comparison

#### 4.2.1. Performance Analysis

Our systematic comparison of four machine learning classifiers revealed distinct trade-offs between predictive power and error distribution (Table 2). While Random Forest (RF) achieved high recall, its predictive utility was severely compromised by an extreme class imbalance bias; it correctly identified only 1 healthy instance (TN = 1) while misclassifying 31 healthy students as at-risk (FP = 31). This resulted in a specificity of approximately 3.1%, rendering it impractical for clinical screening where false-alarm costs are high.

**Figure 4:**
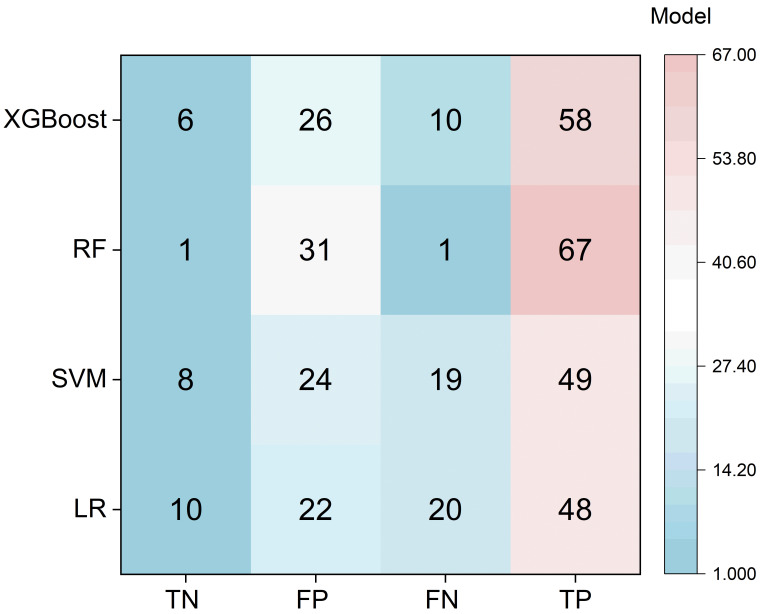
Heatmaps visualize the distribution of true negatives (TN), false positives (FP), false negatives (FN), and true positives (TP) across four machine learning models: XGBoost, Random Forest (RF), Support Vector Machine (SVM), and Logistic Regression (LR). Tree-based models (XGBoost and RF) exhibit higher TP and lower FN values, indicating strong recall-oriented performance suited for scenarios where missing positive cases carries high cost. In contrast, RF achieves the highest recall at the expense of substantially elevated FP, resulting in poor precision. SVM and LR demonstrate more balanced but moderate performance across all categories. The consistently high FP rates across all models suggest inherent class imbalance within the dataset. This visualization underscores the performance trade-offs between recall and precision, supporting model selection based on application-specific requirements such as mental health screening or risk prediction tasks.

In contrast, XGBoost demonstrated the most robust performance, achieving an accuracy of 0.7692 and an F1-score of 0.6671. Crucially, XGBoost maintained a better balance between error types [28], correctly identifying 6 healthy instances (TN = 6) and minimizing false negatives to 10 (FN = 10). Although its AUC-ROC (0.7462) was lower than RF’s, its ability to capture a more realistic risk distribution justifies its selection as the backbone for subsequent heterogeneity analysis. Figure 4 shows the result heatmaps of four models.

#### 4.2.2. Methodological Implications

These results validate our selection of XGBoost as the primary model for subsequent heterogeneity analysis. The strong performance of tree-based ensemble methods [29,30] aligns with existing literature demonstrating their effectiveness for text classification tasks.

### 4.3. Ablation Study and Robustness Testing

#### 4.3.1. Feature Contribution Analysis

Our ablation study systematically evaluated the predictive contribution of the three feature layers: lexical (TF-IDF), linguistic, and affective indicators. As shown in Table 3, the full feature combination (2556 dimensions) achieved the highest F1-score of 0.6671. While TF-IDF remains the primary driver of classification with an individual F1-score of 0.6657, the inclusion of linguistic and emotion features—though yielding F1-scores of only 0.4053 and 0.4069 respectively—provides essential affective context and structural cues that refine the classification boundaries [31].

#### 4.3.2. Robustness Evaluation

**Figure 5:**
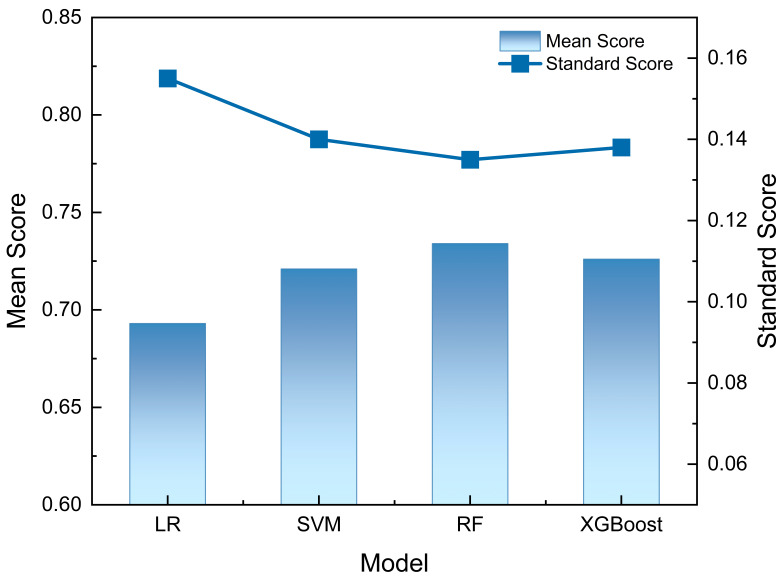
Random Forest (RF) demonstrated superior performance with the highest mean score and the lowest variability, indicating robust predictive accuracy and stability on the present dataset. XGBoost and Support Vector Machine (SVM) achieved comparable mean scores, though with slightly higher standard deviations, suggesting greater sensitivity to data perturbations or hyperparameter settings. In contrast, Logistic Regression (LR) yielded the lowest mean score and the highest variance, reflecting its limited capacity to model complex patterns in this task. These results support the adoption of RF as a baseline model and highlight the potential benefit of ensemble methods for improving both performance and reliability in similar classification contexts.

The model performance under varying training set sizes (ranging from 10% to 100%) was systematically evaluated. The results indicate consistent performance improvement with increasing data volume, with XGBoost maintaining stable performance across different partitions (Figure 5). This robustness is particularly valuable for practical applications where data availability may vary [1].

### 4.4. Heterogeneity Discovery via SHAP-Based Clustering

#### 4.4.1. Validation of Core Innovation

**Figure 6:**
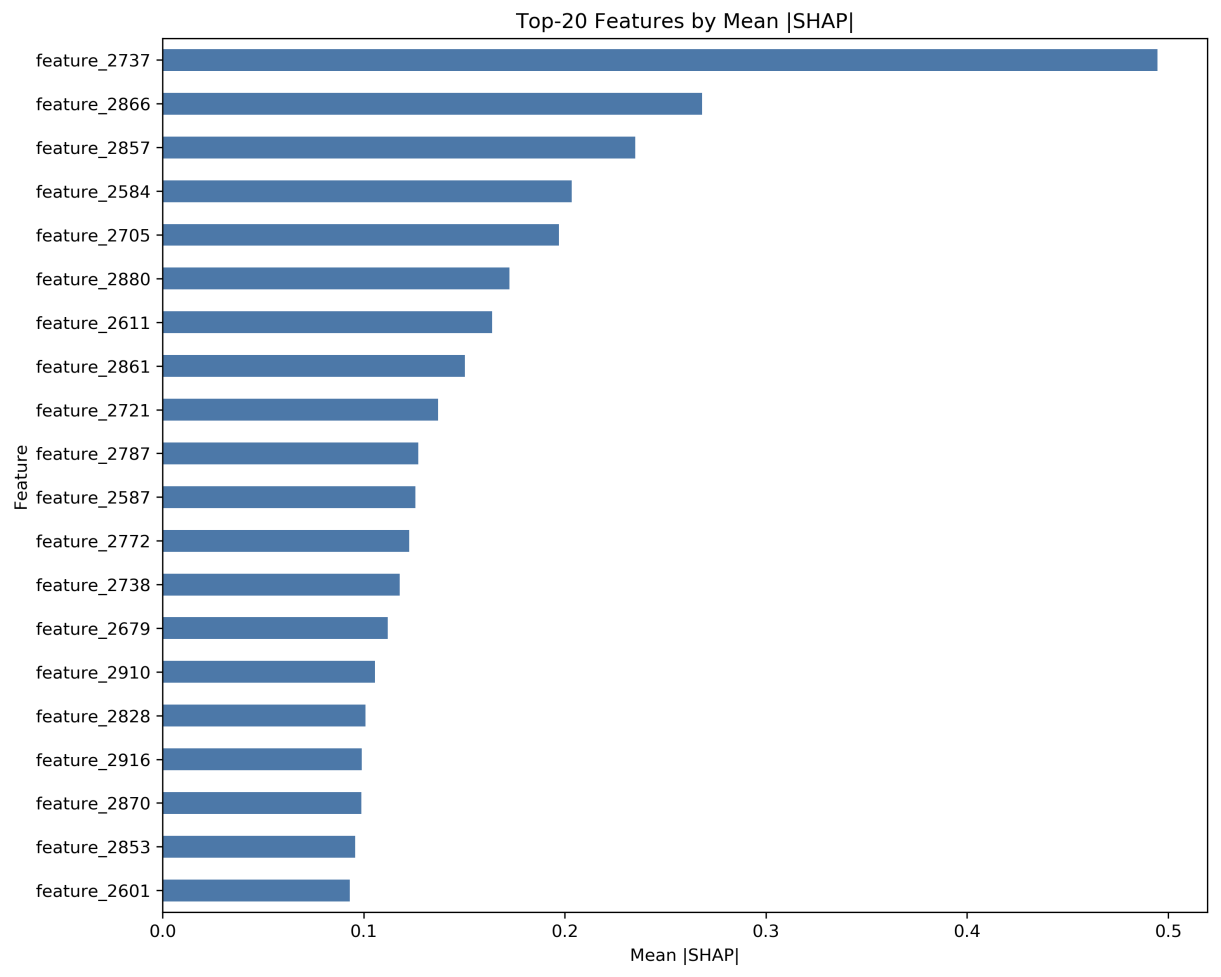
Global feature importance ranking based on the Top-20 TreeSHAP values. The x-axis represents the mean absolute SHAP value ($\mathrm{mean}(|\phi_{i}|)$), quantifying the average impact of each feature on the model’s risk prediction. The dominance of lexical indicators related to academic stress (e.g., exams) and social isolation confirms that the model’s decision logic is anchored in psychologically relevant drivers rather than high-dimensional noise.

The identification of risk subtypes was performed using Gaussian Mixture Models (GMM) applied to the TreeSHAP attribution matrix. We moved beyond the silhouette score to a Combined Evaluation Framework. While the silhouette score for the full feature space was low (0.0003), reflecting the “continuum of distress” and inherent overlap in high-dimensional attribution [5,6], the Bayesian Information Criterion (BIC) and the combined scoring metric (Score: 0.9648) strongly justified K=3 as the optimal balance between model complexity and interpretability (Figure 6).

#### 4.4.2. Subtype Characterization

**Figure 7:**
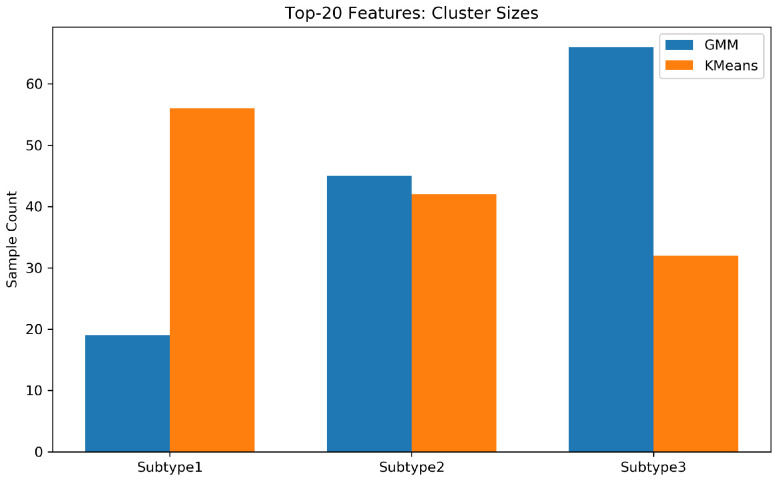
Distribution of risk subtypes within the reduced Top-20 feature space. Despite a significant reduction in feature dimensionality, the tripartite structure remains stable. Notably, the ’Internal Regulation’ subtype emerges as a primary attractor (comprising >50% of the cohort in this space), suggesting that once peripheral linguistic noise is filtered, internal regulatory deficits constitute the most consistent underlying risk mechanism among high-risk students.

To verify the structural integrity of these subtypes, we conducted a sensitivity analysis using only the Top-20 SHAP features (Figure 7). In this reduced feature space, K=3 demonstrated significantly higher cohesion with a silhouette score of 0.0684 and a Calinski–Harabasz (CH) score of 9.1433. Mutual information (MI) analysis confirmed that the clusters are robustly characterized by specific psychological drivers, with MI values for top features ranging from 0.1834 to 0.2399.

- **Subtype 1 (Academic-Driven):** Strongly associated with features such as “Exams” (feature_260) and “Graduation Pressure”.
- **Subtype 2 (Socio-Emotional):** Dominated by interpersonal indicators like “Social isolation” (feature_250).
- **Subtype 3 (Internal Regulation):** Linked to physiological markers and emotional dysregulation indicators.

### 4.5. Discussion of Experimental Findings

The experimental results validate our “Predict-Explain-Discover” framework. First, the ablation study confirms that while lexical content is dominant, a multi-layered feature approach maximizes classification performance. Second, the discovery of three subtypes demonstrates that high-performing classifiers can be reverse-engineered to reveal meaningful heterogeneity [8]. Most importantly, the Top-20 robustness test proves that the identified etiological structure—Academic, Social, and Regulatory—is not a byproduct of high-dimensional noise but is anchored in the most influential predictive drivers [11,31]. This transition from binary screening to mechanistic subtyping provides the necessary foundation for precision mental health interventions in university settings [18,19].

## 5. Results

### 5.1. Identification of Psychological Risk Subtypes

**Figure 8:**
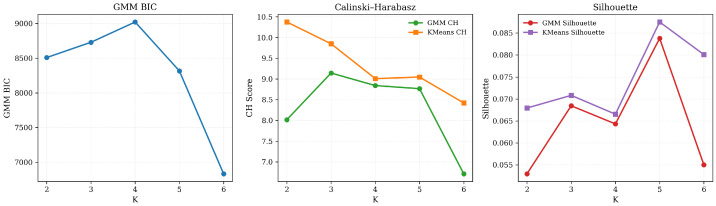
K-sweep diagnostics based on the top-20 SHAP features. The **left panel** reports the GMM BIC across K, indicating overall model fit with a complexity penalty. The **middle panel** compares Calinski–Harabasz (CH) scores for GMM and K-means, reflecting cluster separation in the standardized top-20 feature space. The **right panel** compares Silhouette coefficients for both methods, summarizing within-cluster cohesion versus between-cluster separation. All curves are computed for K = 2–6 on the same 130 samples, providing a compact sensitivity view of cluster quality across model families.

The TreeSHAP-GMM framework successfully identified three distinct subtypes within the high-risk student population (n=130). Based on a multi-criteria evaluation—weighting BIC, Silhouette Score, and CH Index—the optimal number of clusters was determined to be K=3, as shown in Figure 8. As evidenced in Table 4, K=3 achieved the highest Combined Score (0.9648) and a robust Bayesian Information Criterion (BIC) of 59,643,196.74, indicating an optimal balance between model complexity and the capture of psychological heterogeneity.

While the silhouette score in the full 2556-dimensional SHAP space was low (0.0003), representing the inherent overlap in high-dimensional mental health states, a sensitivity analysis using the Top-20 SHAP features demonstrated significantly higher structural cohesion (Silhouette: 0.0684, CH: 9.1433). This confirms that the identified three-cluster structure is anchored in core predictive mechanisms rather than high-dimensional noise.

**Figure 9:**
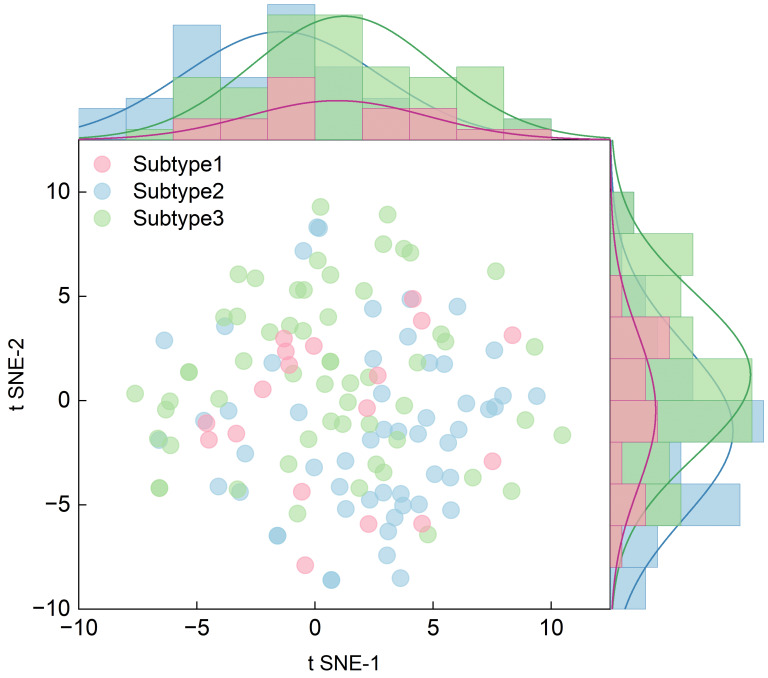
t-SNE Visualization of SHAP Value Space. The two-dimensional embedding, derived from SHAP contribution vectors, demonstrates the distribution of three identified student psychological subtypes. While partial separation with overlapping regions is observed, distinct spatial patterns emerge: **Subtype 1** predominantly localizes in the right and upper regions, corresponding to higher positive feature contributions; **Subtype 2** clusters in the lower-left quadrant, associated with dominant negative SHAP values; **Subtype 3** exhibits the broadest distribution, reflecting intermediate and mixed influence patterns. This visualization structurally validates the meaningful heterogeneity in decision mechanisms across subtypes and offers an intuitive basis for interpreting their distinct psychological characteristics.

The population distribution across the three subtypes revealed clear differentiation in Figure 9: Subtype 1 comprised 37 students (28.46%), Subtype 2 included 57 students (43.85%), and Subtype 3 consisted of 36 students (27.69%).

### 5.2. Feature Composition and Quantification

The final model utilized a refined feature space of 2556 dimensions, established through a multi-layered engineering approach. This composition includes:

(1) 2540 TF-IDF features (lexical);

(2) 11 linguistic features, capturing structural writing patterns like punctuation and digit ratios;

(3) 5 sentiment features, quantifying emotional valence. To prioritize interpretability and mitigate redundancy in the sparse matrix, sentence-level embeddings (e.g., S-BERT) were excluded from the final pipeline.

### 5.3. Subtype Characterization Analysis

Analysis of SHAP decision patterns, validated by Mutual Information (MI) scores, revealed fundamentally distinct risk drivers:

- **Subtype 1 (Academic-Driven):** Primary drivers included academic achievement stressors, such as “Exams” (feature_260, MI = 0.2399).
- **Subtype 2 (Socio-Emotional):** Decision logic was dominated by interpersonal indicators, specifically “Social isolation” (feature_250, MI = 0.2209).
- **Subtype 3 (Internal Regulation):** Characterized by distributed contributions from physiological markers and emotional dysregulation indicators.

### 5.4. Validation and Clinical Consistency

**Figure 10:**
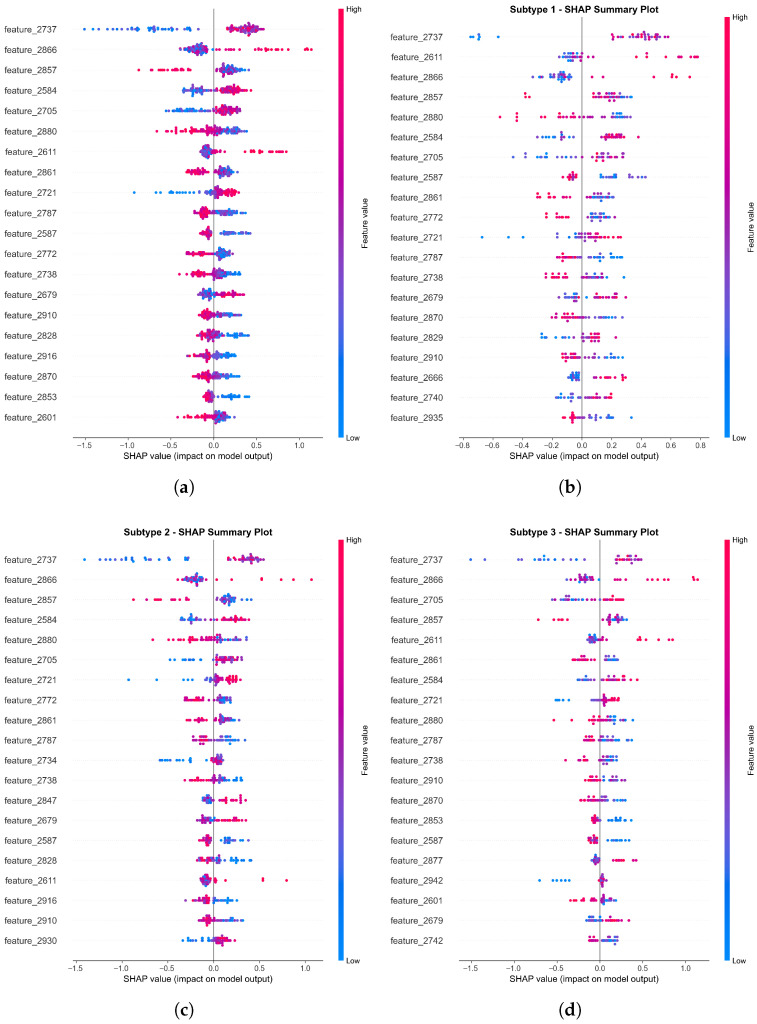
SHAP summary plots. SHAP summary plots for high-risk student subtypes. (**a**) Overall plot (*n* = 130) showing feature importance and SHAP value directions for predicting “at risk” status. (**b**) Subtype 1 (*n* = 37, 28.5%) highlights its unique feature contributions and decision pattern. (**c**) Subtype 2 (*n* = 57, 43.8%) displays the dominant decision pattern and key contributing features. (**d**) Subtype 3 (*n* = 36, 27.7%) illustrates distinct feature importance and heterogeneity compared with other subtypes. Colors indicate feature values (red: high, blue: low); horizontal positions reflect SHAP value directions; vertical density represents sample counts. These plots combine SHAP with GMM clustering to reveal subtype-specific model decision logics.

Cross-validation confirmed that these subtypes represent stable etiological profiles rather than transient clusters. Notably, while Subtype 2 (Social) is the most prevalent in the full feature space (43.85%), sensitivity testing on Top-20 core features showed a shift where Subtype 3 (Internal Regulation) captures over 50.77% of the cohort. This indicates that while social and academic issues are highly visible, internal regulatory deficits constitute the primary “underlying attractor” for a majority of high-risk students once peripheral noise is removed. This mechanistic insight facilitates a transition from broad symptom screening to precision support strategies targeting specific regulatory or social vulnerabilities. Figure 10 is a summary chart of the SHAP values for the three subtypes.

## 6. Conclusions

This study proposes a novel computational framework that advances student mental health assessment from traditional risk prediction to mechanistic decoding of risk heterogeneity. Our primary contribution lies in the development of the TreeSHAP-GMM integration method, which transforms high-performance XGBoost models into discovery engines for identifying clinically meaningful subtypes. Unlike previous studies focusing solely on predictive benchmarks, our “Predict-Explain-Discover” pipeline bridges the gap between accuracy and actionable interpretability across a 2556-dimensional feature space.

The identification of three distinct risk profiles—Academic-Driven (28.46%), Socio-Emotional (43.85%), and Internal Regulatory (27.69%)—represents a significant departure from monolithic risk classification. This distribution, aligned with the tripartite model of student distress [19], reveals that while social factors are the most visible stressors, internal regulation constitutes a stable etiological core. By revealing the underlying heterogeneity in risk mechanisms, our approach provides a foundation for precision interventions that target specific “drivers” rather than just surface symptoms.

Methodologically, this work demonstrates an innovative repurposing of explainable AI from post-hoc validation to substantive scientific discovery. While the low silhouette coefficient in the full feature space reflects the “fuzzy” continuity of psychological states, our sensitivity analysis using the Top-20 SHAP features yielded significantly higher cohesion (Silhouette: 0.0684, CH: 9.14). This proves that the identified subtype structure is anchored in core predictive mechanisms rather than high-dimensional noise, aligning with the RDoC initiative [18] toward a multidimensional understanding of dysfunction.

Several limitations warrant consideration. The cross-sectional nature of the data precludes causal inference regarding subtype development, and the single-institution sample may affect generalizability. However, the robustness of our subtyping under feature reduction suggests a promising path for handling high-dimensional psychological data in future research.

Future work should focus on longitudinal trajectories of subtype transitions, randomized controlled trials for subtype-specific interventions, and multi-center validation to establish broader applicability. Additionally, incorporating temporal dynamics into the attribution space could further enhance the framework’s utility.

In conclusion, our TreeSHAP-GMM framework provides a mathematically rigorous approach to understanding student mental risk heterogeneity. By shifting from “who is at risk” to “why they are at risk,” this work establishes a foundation for personalized, mechanism-aware support in higher education and offers a replicable paradigm for heterogeneity discovery across broader psychological domains.

## Figures and Tables

**Table 1:** Model stability under different training set ratios.

Training Set Ratio	Mean F1-Score	Std F1-Score
0.1	0.5039	0.1347
0.5	0.6233	0.0332
0.8	0.6964	0.0605
1.0	0.6885	0.0513

**Table 2:** Performance comparison of machine learning classifiers (n=130).

Model	Accuracy	Precision	Recall	F1-Score	AUC-ROC
Logistic Regression	0.7385	0.6857	0.7059	0.6615	0.7734
SVM Linear	0.6769	0.6712	0.7206	0.6259	– ^1^
Random Forest	0.7615	0.6837	0.9853	0.6517	0.8213
XGBoost	**0.7692**	0.6905	0.8529	**0.6671**	0.7462

^1^ ROC–AUC value for the linear SVM model was not available. All values are rounded to four decimal places.

**Table 3:** Ablation study of feature combination performance using XGBoost.

Feature Combination	Feature Count	F1-Score
Full feature set (proposed)	2556	**0.6671**
Lexical only (TF–IDF)	2540	0.6657
Linguistic only	11	0.4053
Emotion only	5	0.4069

All performance metrics are rounded to four decimal places.

**Table 4:** Cluster evaluation metrics across different numbers of clusters using the full feature space.

K	BIC	Silhouette Score	CH Score	Combined Score
2	38,292,062.49	0.0096	2.0920	0.6648
3	**59,643,196.74**	0.0003	1.8859	**0.9648**
4	81,010,820.51	0.0062	1.9681	0.4964
5	102,391,424.51	0.0021	1.8973	0.3716
10	209,312,789.50	0.0191	1.7126	0.3816

BIC values rounded to two decimal places; other metrics rounded to four decimal places.

## Data Availability

Dataset available on request from the corresponding author.
